# Bis{μ-4-chloro-*N*′-[(*E*)-1-(5-chloro-2-oxidophen­yl)ethyl­idene]benzohydrazidato}bis­[pyridine­copper(II)]

**DOI:** 10.1107/S1600536811050720

**Published:** 2011-11-30

**Authors:** Jian-Guo Chang

**Affiliations:** a Department of Materials Science and Chemical Engineering, Taishan University, 271021 Taian, Shandong, People’s Republic of China

## Abstract

The crystal structure of the title complex, [Cu_2_(C_15_H_10_Cl_2_N_2_O_2_)_2_(C_5_H_5_N)_2_], features centrosymmetric dimers. The Cu^II^ ion is penta­coordinated in a quadratic pyramidal mode. The quadratic plane is formed by the *O*,*O*′,*N*-tridentate ligand and a pyridine mol­ecule. The fifth coordination site is occupied by the O atom of another ligand showing a significantly longer Cu—O bond.

## Related literature

For further details of the chemistry of the title compound, see: Salem (1998 ▶). For a related structure, see: Chang (2008 ▶).
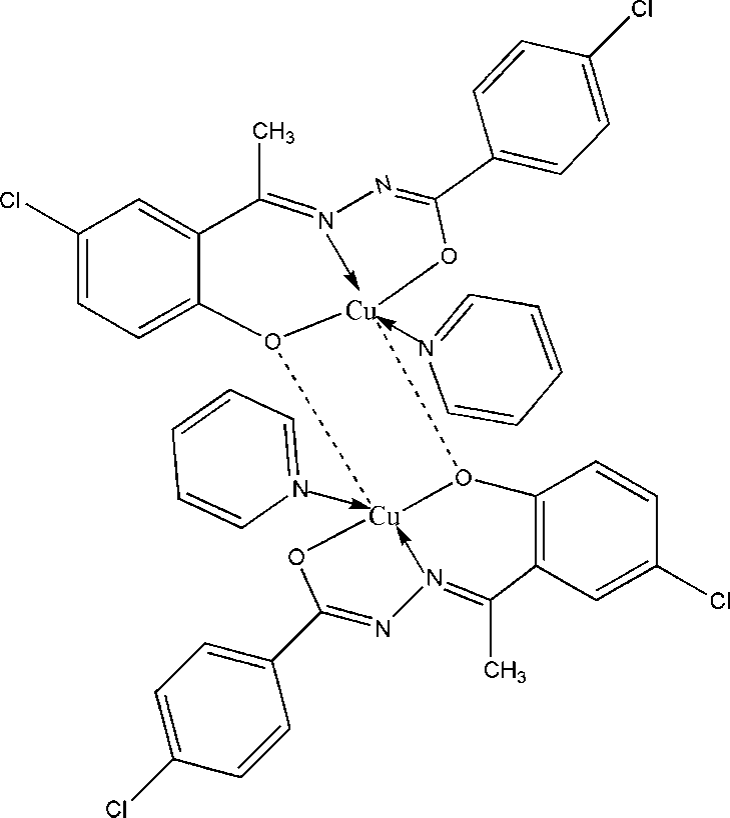


## Experimental

### 

#### Crystal data

[Cu_2_(C_15_H_10_Cl_2_N_2_O_2_)_2_(C_5_H_5_N)_2_]
*M* *_r_* = 927.58
Monoclinic, 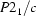

*a* = 11.913 (2) Å
*b* = 8.0783 (16) Å
*c* = 19.997 (4) Å
β = 95.66 (3)°
*V* = 1915.1 (7) Å^3^
*Z* = 2
Mo *K*α radiation
μ = 1.44 mm^−1^
*T* = 298 K
0.28 × 0.25 × 0.18 mm

#### Data collection

Bruker APEXII CCD area-detector diffractometer
Absorption correction: multi-scan (*SADABS*; Sheldrick, 2003 ▶) *T*_min_ = 0.688, *T*_max_ = 0.782
9685 measured reflections
3411 independent reflections
2976 reflections with *I* > 2σ(*I*)
*R*_int_ = 0.023

#### Refinement

*R*[*F* ^2^ > 2σ(*F* ^2^)] = 0.026
*wR*(*F* ^2^) = 0.064
*S* = 1.02
3411 reflections
255 parameters
H-atom parameters constrained
Δρ_max_ = 0.22 e Å^−3^
Δρ_min_ = −0.23 e Å^−3^

### 

Data collection: *APEX2* (Bruker, 2005 ▶); cell refinement: *SAINT* (Bruker, 2005 ▶); data reduction: *SAINT*; program(s) used to solve structure: *SHELXS97* (Sheldrick, 2008 ▶); program(s) used to refine structure: *SHELXL97* (Sheldrick, 2008 ▶); molecular graphics: *SHELXTL* (Sheldrick, 2008 ▶); software used to prepare material for publication: *SHELXTL*.

## Supplementary Material

Crystal structure: contains datablock(s) global, I. DOI: 10.1107/S1600536811050720/bt5720sup1.cif

Structure factors: contains datablock(s) I. DOI: 10.1107/S1600536811050720/bt5720Isup2.hkl

Additional supplementary materials:  crystallographic information; 3D view; checkCIF report

## Figures and Tables

**Table 1:** Selected bond lengths (Å)

Cu1—O2	1.8824 (14)
Cu1—O1	1.9209 (15)
Cu1—N2	1.9475 (17)
Cu1—N3	2.0275 (17)
Cu1—O2^i^	2.6055 (16)

Symmetry code: (i) 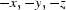.

**Fig. 1.**
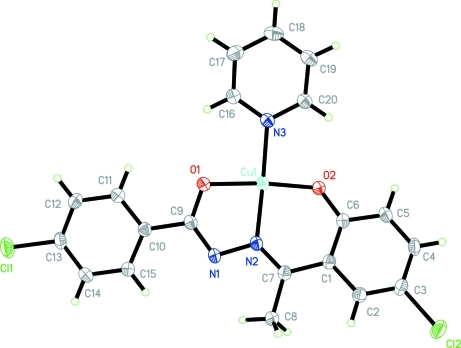
The molecular structure of compound (I). Displacement ellipsoids are drawn at the 30% probability level.

**Fig. 2.**
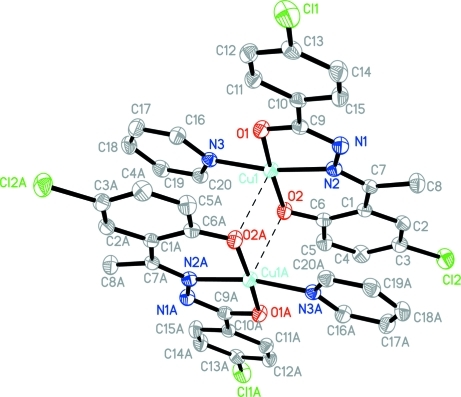
The dimeric structure of the title compound . H atoms have been omitted for clarity. [Dashed lines show Cu—O weak interactions].

## supplementary crystallographic information

### Comment

The chemistry of aroylhydrazones has gained a special attraction due to their coordination abilities to metal ions (Salem, 1998). As an extension of work on the structural characterization of aroylhydrazone derivatives (Chang, 2008), the title compound, (I), was synthesized and its crystal structure is reported here.

The new complex, (I), the Cu II ion exhibits a distorted *trans*-Cu_2_O_2_ square-planar geometry arising from the O, O, N-tridentate ligand and a pyridine molecule. (see Fig. 1).Two molecules form a weak-bridged dimer with weak Cu—O interactions. (see Fig. 2).

### Experimental

The ligand 4-chloro-*N*'-[(1E)-1-(5-chloro-2-hydroxyphenyl)ethylidene]benzohydrazide was prepared by the reaction of 1-(5-chloro-2-hydroxyphenyl)ethanone and 4-chlorobenzohydrazide in a molar ratio of 1:1 under reflux in ethanol for 4 h. The white precipitate was collected, washed several times with ethanol and dried *in vacuo* (yield 83%).A DMF solution (5 ml) of the ligand (0.25 mmol, 0.081 g) was mixed with a methanol solution(5 ml) of Cu(OAc)2 (0.25 mmol, 0.05 g). The mixture was stirred at 298 K for 4 h and then filtered. A blue precipitate was produced after about 10 d. A pyridine mixture (5 ml) was used to dissolve the precipitate at 330 K. Blue block-shaped crystals were obtained after one month (yield 25%).

### Refinement

All H atoms were positioned geometrically and treated as riding on their parent atoms,with *C*—*H*(methyl) = 0.96 Å, C—H(aromatic) = 0.93 Å and with *U*_iso_(H) =1.5*U*_eq_(C_methyl_) and 1.2*U*_eq_(C_aromatic_).

### Crystal data

**Table:**

[Cu_2_(C_15_H_10_Cl_2_N_2_O_2_)_2_(C_5_H_5_N)_2_]	*F*(000) = 940
*M**_r_* = 927.58	*D*_x_ = 1.609 Mg m^−^^3^
Monoclinic, *P*2_1_/*c*	Mo *K*α radiation, λ = 0.71073 Å
Hall symbol: -P 2ybc	Cell parameters from 4831 reflections
*a* = 11.913 (2) Å	θ = 2.5–27.9°
*b* = 8.0783 (16) Å	µ = 1.44 mm^−^^1^
*c* = 19.997 (4) Å	*T* = 298 K
β = 95.66 (3)°	Block, blue
*V* = 1915.1 (7) Å^3^	0.28 × 0.25 × 0.18 mm
*Z* = 2	

### Data collection

**Table:**

Bruker APEXII CCD area-detector diffractometer	3411 independent reflections
Radiation source: fine-focus sealed tube	2976 reflections with *I* > 2σ(*I*)
graphite	*R*_int_ = 0.023
φ and ω scans	θ_max_ = 25.1°, θ_min_ = 2.1°
Absorption correction: multi-scan (*SADABS*; Sheldrick, 2003)	*h* = −13→14
*T*_min_ = 0.688, *T*_max_ = 0.782	*k* = −9→9
9685 measured reflections	*l* = −22→23

### Refinement

**Table:**

Refinement on *F*^2^	Secondary atom site location: difference Fourier map
Least-squares matrix: full	Hydrogen site location: inferred from neighbouring sites
*R*[*F*^2^ > 2σ(*F*^2^)] = 0.026	H-atom parameters constrained
*wR*(*F*^2^) = 0.064	*w* = 1/[σ^2^(*F*_o_^2^) + (0.0242*P*)^2^ + 1.1862*P*] where *P* = (*F*_o_^2^ + 2*F*_c_^2^)/3
*S* = 1.02	(Δ/σ)_max_ < 0.001
3411 reflections	Δρ_max_ = 0.22 e Å^−^^3^
255 parameters	Δρ_min_ = −0.23 e Å^−^^3^
0 restraints	Extinction correction: *SHELXL97* (Sheldrick, 2008), Fc^*^=kFc[1+0.001xFc^2^λ^3^/sin(2θ)]^-1/4^
Primary atom site location: structure-invariant direct methods	Extinction coefficient: 0.0011 (3)

### Special details

**Table:**

Geometry. All e.s.d.'s (except the e.s.d. in the dihedral angle between two l.s. planes) are estimated using the full covariance matrix. The cell e.s.d.'s are taken into account individually in the estimation of e.s.d.'s in distances, angles and torsion angles; correlations between e.s.d.'s in cell parameters are only used when they are defined by crystal symmetry. An approximate (isotropic) treatment of cell e.s.d.'s is used for estimating e.s.d.'s involving l.s. planes.
Refinement. Refinement of *F*^2^ against ALL reflections. The weighted *R*-factor *wR* and goodness of fit *S* are based on *F*^2^, conventional *R*-factors *R* are based on *F*, with *F* set to zero for negative *F*^2^. The threshold expression of *F*^2^ > σ(*F*^2^) is used only for calculating *R*-factors(gt) *etc*. and is not relevant to the choice of reflections for refinement. *R*-factors based on *F*^2^ are statistically about twice as large as those based on *F*, and *R*- factors based on ALL data will be even larger.

### Fractional atomic coordinates and isotropic or equivalent isotropic displacement parameters (Å^2^)

**Table:**

	*x*	*y*	*z*	*U*_iso_*/*U*_eq_	
Cu1	0.09353 (2)	0.14880 (3)	0.017875 (12)	0.03476 (10)	
Cl1	0.49728 (6)	0.08946 (11)	0.39268 (3)	0.0677 (2)	
Cl2	0.23787 (6)	−0.18478 (10)	−0.29800 (3)	0.0638 (2)	
C1	0.19626 (16)	−0.0360 (3)	−0.10969 (10)	0.0334 (4)	
C2	0.24154 (18)	−0.0995 (3)	−0.16734 (10)	0.0389 (5)	
H2	0.3158	−0.1368	−0.1638	0.047*	
C13	0.43005 (18)	0.0975 (3)	0.31108 (10)	0.0425 (5)	
C6	0.08344 (17)	0.0220 (3)	−0.11671 (10)	0.0349 (5)	
C9	0.26194 (17)	0.1119 (3)	0.11379 (10)	0.0355 (5)	
C10	0.32367 (17)	0.1101 (3)	0.18203 (10)	0.0350 (5)	
C7	0.26861 (16)	−0.0379 (3)	−0.04553 (10)	0.0340 (4)	
C3	0.17827 (19)	−0.1071 (3)	−0.22803 (10)	0.0425 (5)	
C14	0.47580 (18)	0.0123 (3)	0.26053 (11)	0.0496 (6)	
H14	0.5416	−0.0489	0.2696	0.060*	
C12	0.33351 (19)	0.1898 (3)	0.29860 (11)	0.0439 (5)	
H12	0.3045	0.2480	0.3331	0.053*	
C16	−0.04746 (19)	0.3803 (3)	0.08770 (11)	0.0442 (5)	
H16	0.0054	0.3580	0.1239	0.053*	
C11	0.27997 (18)	0.1952 (3)	0.23412 (11)	0.0410 (5)	
H11	0.2140	0.2564	0.2254	0.049*	
C15	0.42230 (18)	0.0195 (3)	0.19631 (11)	0.0457 (6)	
H15	0.4527	−0.0373	0.1619	0.055*	
C5	0.02189 (19)	0.0115 (3)	−0.18052 (11)	0.0447 (5)	
H5	−0.0523	0.0491	−0.1857	0.054*	
C17	−0.1338 (2)	0.4880 (3)	0.09677 (13)	0.0531 (6)	
H17	−0.1394	0.5365	0.1385	0.064*	
C4	0.0679 (2)	−0.0524 (3)	−0.23534 (11)	0.0472 (6)	
H4	0.0254	−0.0587	−0.2768	0.057*	
C8	0.38055 (18)	−0.1255 (3)	−0.04096 (11)	0.0470 (6)	
H8A	0.4069	−0.1434	0.0054	0.071*	
H8B	0.4341	−0.0589	−0.0617	0.071*	
H8C	0.3720	−0.2301	−0.0637	0.071*	
C19	−0.20175 (18)	0.4478 (3)	−0.01709 (12)	0.0477 (6)	
H19	−0.2536	0.4692	−0.0540	0.057*	
C18	−0.21219 (19)	0.5235 (3)	0.04330 (13)	0.0515 (6)	
H18	−0.2709	0.5971	0.0481	0.062*	
C20	−0.11366 (17)	0.3397 (3)	−0.02264 (11)	0.0401 (5)	
H20	−0.1077	0.2882	−0.0637	0.048*	
O2	0.02859 (12)	0.07997 (19)	−0.06722 (7)	0.0410 (4)	
O1	0.16834 (12)	0.19404 (19)	0.10550 (7)	0.0408 (4)	
N2	0.23464 (13)	0.0347 (2)	0.00719 (8)	0.0347 (4)	
N1	0.30604 (14)	0.0264 (2)	0.06694 (8)	0.0389 (4)	
N3	−0.03615 (14)	0.3059 (2)	0.02877 (8)	0.0346 (4)	

### Atomic displacement parameters (Å^2^)

**Table:**

	*U*^11^	*U*^22^	*U*^33^	*U*^12^	*U*^13^	*U*^23^
Cu1	0.03293 (15)	0.03761 (16)	0.03257 (15)	0.00326 (11)	−0.00261 (10)	−0.00316 (11)
Cl1	0.0542 (4)	0.1114 (6)	0.0349 (3)	−0.0007 (4)	−0.0088 (3)	−0.0007 (3)
Cl2	0.0581 (4)	0.0963 (5)	0.0374 (3)	−0.0031 (4)	0.0078 (3)	−0.0195 (3)
C1	0.0347 (11)	0.0345 (11)	0.0307 (10)	−0.0046 (9)	0.0023 (8)	0.0004 (9)
C2	0.0362 (11)	0.0438 (13)	0.0367 (11)	−0.0040 (9)	0.0041 (9)	−0.0007 (10)
C13	0.0376 (12)	0.0582 (14)	0.0304 (11)	−0.0076 (10)	−0.0039 (9)	0.0000 (10)
C6	0.0381 (11)	0.0345 (11)	0.0316 (11)	−0.0027 (9)	0.0009 (9)	0.0012 (9)
C9	0.0335 (11)	0.0367 (12)	0.0353 (11)	−0.0029 (9)	−0.0015 (9)	−0.0004 (9)
C10	0.0330 (10)	0.0374 (12)	0.0341 (11)	−0.0039 (9)	0.0007 (9)	−0.0034 (9)
C7	0.0329 (10)	0.0356 (11)	0.0336 (11)	−0.0051 (9)	0.0030 (8)	0.0001 (9)
C3	0.0475 (13)	0.0507 (14)	0.0298 (11)	−0.0068 (10)	0.0068 (9)	−0.0030 (10)
C14	0.0348 (12)	0.0676 (17)	0.0445 (13)	0.0090 (11)	−0.0053 (10)	−0.0052 (12)
C12	0.0475 (13)	0.0497 (14)	0.0349 (12)	0.0007 (11)	0.0062 (10)	−0.0066 (10)
C16	0.0432 (12)	0.0481 (14)	0.0404 (13)	0.0050 (10)	−0.0004 (10)	−0.0030 (10)
C11	0.0378 (12)	0.0446 (13)	0.0400 (12)	0.0041 (10)	0.0008 (9)	−0.0037 (10)
C15	0.0376 (12)	0.0615 (15)	0.0372 (12)	0.0080 (11)	−0.0004 (9)	−0.0107 (11)
C5	0.0394 (12)	0.0570 (15)	0.0360 (12)	0.0051 (11)	−0.0046 (9)	0.0027 (11)
C17	0.0502 (14)	0.0567 (16)	0.0528 (14)	0.0076 (12)	0.0071 (11)	−0.0121 (12)
C4	0.0502 (14)	0.0619 (16)	0.0281 (11)	−0.0018 (11)	−0.0039 (10)	0.0026 (11)
C8	0.0367 (12)	0.0669 (16)	0.0366 (12)	0.0067 (11)	−0.0011 (9)	−0.0077 (11)
C19	0.0350 (12)	0.0509 (14)	0.0557 (15)	0.0033 (10)	−0.0034 (10)	0.0052 (12)
C18	0.0365 (12)	0.0491 (14)	0.0697 (17)	0.0082 (11)	0.0092 (11)	−0.0007 (13)
C20	0.0369 (11)	0.0402 (12)	0.0424 (12)	−0.0003 (9)	0.0003 (9)	0.0013 (10)
O2	0.0348 (8)	0.0523 (9)	0.0348 (8)	0.0070 (7)	−0.0024 (6)	−0.0080 (7)
O1	0.0370 (8)	0.0468 (9)	0.0370 (8)	0.0084 (7)	−0.0043 (6)	−0.0071 (7)
N2	0.0331 (9)	0.0400 (10)	0.0298 (9)	−0.0012 (7)	−0.0028 (7)	−0.0018 (8)
N1	0.0337 (9)	0.0506 (11)	0.0309 (9)	0.0029 (8)	−0.0041 (7)	−0.0044 (8)
N3	0.0324 (9)	0.0340 (9)	0.0370 (10)	−0.0005 (7)	0.0005 (7)	0.0014 (8)

### Geometric parameters (Å, °)

**Table:**

Cu1—O2	1.8824 (14)	C14—C15	1.377 (3)
Cu1—O1	1.9209 (15)	C14—H14	0.9300
Cu1—N2	1.9475 (17)	C12—C11	1.382 (3)
Cu1—N3	2.0275 (17)	C12—H12	0.9300
Cu1—O2^i^	2.6055 (16)	C16—N3	1.342 (3)
Cl1—C13	1.747 (2)	C16—C17	1.373 (3)
Cl2—C3	1.747 (2)	C16—H16	0.9300
C1—C2	1.417 (3)	C11—H11	0.9300
C1—C6	1.417 (3)	C15—H15	0.9300
C1—C7	1.473 (3)	C5—C4	1.374 (3)
C2—C3	1.365 (3)	C5—H5	0.9300
C2—H2	0.9300	C17—C18	1.379 (3)
C13—C12	1.373 (3)	C17—H17	0.9300
C13—C14	1.379 (3)	C4—H4	0.9300
C6—O2	1.324 (2)	C8—H8A	0.9600
C6—C5	1.410 (3)	C8—H8B	0.9600
C9—O1	1.294 (2)	C8—H8C	0.9600
C9—N1	1.315 (3)	C19—C18	1.370 (3)
C9—C10	1.485 (3)	C19—C20	1.378 (3)
C10—C15	1.390 (3)	C19—H19	0.9300
C10—C11	1.391 (3)	C18—H18	0.9300
C7—N2	1.305 (3)	C20—N3	1.341 (3)
C7—C8	1.504 (3)	C20—H20	0.9300
C3—C4	1.381 (3)	N2—N1	1.398 (2)
			
O2—Cu1—O1	173.26 (6)	C12—C11—C10	120.7 (2)
O2—Cu1—N2	92.48 (7)	C12—C11—H11	119.7
O1—Cu1—N2	82.09 (7)	C10—C11—H11	119.7
O2—Cu1—N3	91.86 (7)	C14—C15—C10	121.2 (2)
O1—Cu1—N3	94.17 (7)	C14—C15—H15	119.4
N2—Cu1—N3	169.49 (7)	C10—C15—H15	119.4
C2—C1—C6	118.22 (18)	C4—C5—C6	122.1 (2)
C2—C1—C7	117.89 (18)	C4—C5—H5	118.9
C6—C1—C7	123.88 (18)	C6—C5—H5	118.9
C3—C2—C1	121.2 (2)	C16—C17—C18	119.2 (2)
C3—C2—H2	119.4	C16—C17—H17	120.4
C1—C2—H2	119.4	C18—C17—H17	120.4
C12—C13—C14	121.5 (2)	C5—C4—C3	119.1 (2)
C12—C13—Cl1	119.26 (17)	C5—C4—H4	120.4
C14—C13—Cl1	119.23 (18)	C3—C4—H4	120.4
O2—C6—C5	116.53 (18)	C7—C8—H8A	109.5
O2—C6—C1	125.16 (18)	C7—C8—H8B	109.5
C5—C6—C1	118.24 (19)	H8A—C8—H8B	109.5
O1—C9—N1	125.31 (18)	C7—C8—H8C	109.5
O1—C9—C10	117.72 (18)	H8A—C8—H8C	109.5
N1—C9—C10	116.95 (18)	H8B—C8—H8C	109.5
C15—C10—C11	118.53 (19)	C18—C19—C20	119.3 (2)
C15—C10—C9	121.73 (19)	C18—C19—H19	120.3
C11—C10—C9	119.68 (19)	C20—C19—H19	120.3
N2—C7—C1	119.81 (18)	C19—C18—C17	118.5 (2)
N2—C7—C8	120.39 (18)	C19—C18—H18	120.7
C1—C7—C8	119.80 (18)	C17—C18—H18	120.7
C2—C3—C4	121.1 (2)	N3—C20—C19	122.7 (2)
C2—C3—Cl2	119.72 (18)	N3—C20—H20	118.7
C4—C3—Cl2	119.21 (17)	C19—C20—H20	118.7
C15—C14—C13	118.8 (2)	C6—O2—Cu1	126.23 (13)
C15—C14—H14	120.6	C9—O1—Cu1	109.62 (13)
C13—C14—H14	120.6	C7—N2—N1	117.24 (16)
C13—C12—C11	119.2 (2)	C7—N2—Cu1	129.88 (14)
C13—C12—H12	120.4	N1—N2—Cu1	112.82 (12)
C11—C12—H12	120.4	C9—N1—N2	109.36 (16)
N3—C16—C17	122.8 (2)	C20—N3—C16	117.44 (18)
N3—C16—H16	118.6	C20—N3—Cu1	121.31 (14)
C17—C16—H16	118.6	C16—N3—Cu1	121.24 (14)
			
C6—C1—C2—C3	−0.8 (3)	C18—C19—C20—N3	−0.6 (3)
C7—C1—C2—C3	177.68 (19)	C5—C6—O2—Cu1	−166.71 (15)
C2—C1—C6—O2	177.84 (19)	C1—C6—O2—Cu1	16.1 (3)
C7—C1—C6—O2	−0.6 (3)	N2—Cu1—O2—C6	−17.20 (17)
C2—C1—C6—C5	0.7 (3)	N3—Cu1—O2—C6	153.23 (17)
C7—C1—C6—C5	−177.69 (19)	N1—C9—O1—Cu1	7.5 (3)
O1—C9—C10—C15	177.2 (2)	C10—C9—O1—Cu1	−170.89 (14)
N1—C9—C10—C15	−1.3 (3)	N2—Cu1—O1—C9	−7.65 (14)
O1—C9—C10—C11	0.0 (3)	N3—Cu1—O1—C9	−177.77 (14)
N1—C9—C10—C11	−178.6 (2)	C1—C7—N2—N1	178.98 (17)
C2—C1—C7—N2	172.96 (19)	C8—C7—N2—N1	−0.2 (3)
C6—C1—C7—N2	−8.6 (3)	C1—C7—N2—Cu1	1.9 (3)
C2—C1—C7—C8	−7.8 (3)	C8—C7—N2—Cu1	−177.31 (16)
C6—C1—C7—C8	170.6 (2)	O2—Cu1—N2—C7	8.57 (19)
C1—C2—C3—C4	0.3 (3)	O1—Cu1—N2—C7	−175.44 (19)
C1—C2—C3—Cl2	179.44 (17)	N3—Cu1—N2—C7	−105.7 (4)
C12—C13—C14—C15	−0.6 (4)	O2—Cu1—N2—N1	−168.59 (13)
Cl1—C13—C14—C15	−179.93 (19)	O1—Cu1—N2—N1	7.40 (13)
C14—C13—C12—C11	1.1 (4)	N3—Cu1—N2—N1	77.1 (4)
Cl1—C13—C12—C11	−179.58 (18)	O1—C9—N1—N2	−1.4 (3)
C13—C12—C11—C10	−0.8 (3)	C10—C9—N1—N2	176.98 (16)
C15—C10—C11—C12	0.0 (3)	C7—N2—N1—C9	176.99 (18)
C9—C10—C11—C12	177.4 (2)	Cu1—N2—N1—C9	−5.5 (2)
C13—C14—C15—C10	−0.2 (4)	C19—C20—N3—C16	0.8 (3)
C11—C10—C15—C14	0.5 (3)	C19—C20—N3—Cu1	−178.99 (17)
C9—C10—C15—C14	−176.8 (2)	C17—C16—N3—C20	−0.2 (3)
O2—C6—C5—C4	−177.5 (2)	C17—C16—N3—Cu1	179.63 (18)
C1—C6—C5—C4	−0.1 (3)	O2—Cu1—N3—C20	−10.18 (16)
N3—C16—C17—C18	−0.6 (4)	O1—Cu1—N3—C20	172.84 (16)
C6—C5—C4—C3	−0.5 (4)	N2—Cu1—N3—C20	104.2 (4)
C2—C3—C4—C5	0.4 (4)	O2—Cu1—N3—C16	170.03 (17)
Cl2—C3—C4—C5	−178.78 (19)	O1—Cu1—N3—C16	−6.95 (17)
C20—C19—C18—C17	−0.2 (4)	N2—Cu1—N3—C16	−75.6 (4)
C16—C17—C18—C19	0.8 (4)		

Symmetry codes: (i) −*x*, −*y*, −*z*.
